# Characterization and frequency of a newly identified HIV-1 BF1 intersubtype circulating recombinant form in São Paulo, Brazil

**DOI:** 10.1186/1743-422X-7-74

**Published:** 2010-04-16

**Authors:** Sabri Saeed Sanabani, Évelyn Regina de Souza Pastena, Walter Kleine Neto, Vanessa Pouza Martinez, Ester Cerdeira Sabino

**Affiliations:** 1 Fundação Pro-Sangue, Hemocentro, São Paulo, Brazil; 2 Retrovirology Laboratory, Federal University of São Paulo, Brazil

## Abstract

**Background:**

HIV circulating recombinant forms (CRFs) play an important role in the global and regional HIV epidemics, particularly in regions where multiple subtypes are circulating. To date, several (>40) CRFs are recognized worldwide with five currently circulating in Brazil. Here, we report the characterization of near full-length genome sequences (NFLG) of six phylogenetically related HIV-1 BF1 intersubtype recombinants (five from this study and one from other published sequences) representing CRF46_BF1.

**Methods:**

Initially, we selected 36 samples from 888 adult patients residing in São Paulo who had previously been diagnosed as being infected with subclade F1 based on *pol *subgenomic fragment sequencing. Proviral DNA integrated in peripheral blood mononuclear cells (PBMC) was amplified from the purified genomic DNA of all 36-blood samples by five overlapping PCR fragments followed by direct sequencing. Sequence data were obtained from the five fragments that showed identical genomic structure and phylogenetic trees were constructed and compared with previously published sequences. Genuine subclade F1 sequences and any other sequences that exhibited unique mosaic structures were omitted from further analysis

**Results:**

Of the 36 samples analyzed, only six sequences, inferred from the *pol *region as subclade F1, displayed BF1 identical mosaic genomes with a single intersubtype breakpoint identified at the *nef*-U3 overlap (HXB2 position 9347-9365; LTR region). Five of these isolates formed a rigid cluster in phylogentic trees from different subclade F1 fragment regions, which we can now designate as CRF46_BF1. According to our estimate, the new CRF accounts for 0.56% of the HIV-1 circulating strains in São Paulo. Comparison with previously published sequences revealed an additional five isolates that share an identical mosaic structure with those reported in our study. Despite sharing a similar recombinant structure, only one sequence appeared to originate from the same CRF46_BF1 ancestor.

**Conclusion:**

We identified a new circulating recombinant form with a single intersubtype breakpoint identified at the *nef*-LTR U3 overlap and designated CRF46_BF1. Given the biological importance of the LTR U3 region, intersubtype recombination in this region could play an important role in HIV evolution with critical consequences for the development of efficient genetic vaccines.

## Background

The immense genetic variability of HIV-1 viruses is considered the key factor that frustrates efforts to halt the virus epidemic and poses a serious challenge to the development and efficacy of vaccines. Like other human positive-sense RNA viruses, HIV has a high mutation rate as a result of the error-prone nature of their reverse transcriptase (3 × 10^-5 ^mutations per nucleotide per replication cycle)[1,2]. This high rate of mutation coupled with the increased replication capacity of the virus (10.3 × 10^9 ^particles per day) [3], allows for the accumulation and fixation of a variety of advantageous genetic changes in a virus population, which are selected for by the host immune response and can resist newly evolving host defense. Recombination is another potential evolutionary source that significantly contributes to the genetic diversification of HIV by successfully repairing defective viral genes and by producing new viruses [4]. To date, HIV-1 viruses are classified into four phylogenetic groups: M, O, N and P, which most likely reflect four independent events of cross-species transmission from chimpanzees [5-7]. The M group (for main), responsible for the majority of viral infection worldwide, is further subdivided into nine subtypes (A-D, F-H, J and K), among which subtypes A and F have been further classified into two sub-subtypes [5]. Moreover, early sequencing studies have provided evidence of recombination between genomes of different HIV subtypes [8,9]. Such interclade recombinant strains are consistently reported from regions where two or more clades are predominant. Recombinant strains from at least three unlinked epidemiological sources, which exhibit identical mosaic patterns, have been classified separately as circulating recombinant forms (CRFs) [10,11]. Currently, there are more than 40 defined CRFs http://www.hiv.lanl.gov that are epidemiologically important as subtypes [12]. In addition to the known CRFs, a large number of unique recombinant viruses, which are called unique recombinant forms (URFs), have been characterized worldwide [13]. Together, CRFs and URFs account for 18% of incident infections in the global HIV-1 pandemic [12]. HIV-1 subtypes, CRFs and URFs show considerably different patterns of distribution in different geographical regions [12,14].

In Brazil, the number of persons living with HIV reached an estimated number of 730,000 cases at the beginning of 2008 (2008 Report on the Global AIDS Epidemic). Like in other European countries and in North America, HIV-1 subtype B is a major genetic clade circulating in the country. However, the existence of other subtypes such as F1, C, B/C and B/F, has been consistently reported [15-23]. Data from recent studies of the near full length genomes (NFLG) of HIV have provided evidence of Brazilian CRF strains designated as CRF28_BF, CRF29_BF, CRF39_BF, CRF40_BF and CRF31_BC [17,24-26]http://www.hiv.lanl.gov/content/sequence/HIV/CRFs/CRFs.html.

In 2006, Thompson and colleagues [27] published two NFLG of similar BF1 mosaic viruses from patients in Rio de Janeiro 94BR-RJ-41 (GenBank: AY455781) and 99UFRJ-16 (GenBank: AY455782). Here, we describe the HIV-1 NFLG of an additional six isolates with similar BF1 mosaic genomes from patients without evidence of direct epidemiological linkage.

## Methods

### Study population

The six samples reported in this study were from individuals residing in São Paulo in the southeast region of Brazil and considered the most populous city in South America. The rationale for selection of these samples has been previously reported [28]. The data, including age, gender, number of CD4-positive T cells, and viral load were obtained from medical records and shown in Table 1. No evidence of direct epidemiological linkage could be established.

**Table 1:** Characteristics of the six patients included in this study.

*Sample ID*	*Age/years*	*Sex*	*CD4 count, cells/mm*^2^	*Viral load, copies/mL*
06BR_FPS561	44	F^1^	81	721
07BR_FPS625	35	F	621	4734
07BR_FPS742	47	F	140	123617
07BR_FPS783	42	M^2^	209	80751
07BR_FPS810	38	F	208	49240

07BR_FPS812	45	F	362	5694

F; Female, M; Male

### Amplification and sequencing of HIV-1 DNA

The genomic DNA used for the PCR analyses was extracted using the QIAamp blood kit (Qiagen) according to the manufacturer instructions. The NFLGs from five overlapping fragments were obtained by PCR using the Platinum *Taq *DNA polymerase (5 U/μl) (Invitrogen) and determined by a previously reported method [16,17]. To rule out the possibility of *Taq*-generated recombinants, an additional PCR product of 670 bp, which spans most of the viral LTR, was generated in separate PCR reactions using previously described primers and conditions [29]. All amplification reactions were done in duplicate to eliminate PCR artifacts, ensuring that sequenced NFLG were not assembled from heterogeneous DNA targets. To test for PCR carry over contamination, extraction and PCR negatives were run in each experiment. Both complementary DNA strands from each amplicon were directly sequenced by cycle sequencing using a variety of internal primers, BigDye terminator chemistry and *Taq *polymerase on an automated sequencer (ABI 3130, Applied Biosystems Inc., Foster City, CA), essentially according to the protocols recommended by the manufacturer. Fragments for each amplicon were assembled into contiguous sequences on a minimum overlap of 30 bp with a 97-100% minimal mismatch and edited using the Sequencher program 4.7 (Gene Code Corp., Ann Arbor, MI).

### Screening for recombination events and identification of breakpoints

Sequences were screened for the presence of recombination patterns by the jumping profile Hidden Markov Model (jpHMM) [30] and further confirmed using the bootscanning method [31] implemented by SimPlot 3.5.1 for Windows [32]. The following parameters were used in this method: window size, 250 bp; step size, 20 bp; the F84 model of evolution (Maximum likelihood (ML)) as a model to estimate nucleotide substitution; transition\transversion ratio, 2.0; and a bootstrap of 100 trees. In addition, the significant threshold for the bootscan was set at 90%. The alignment of multiple sequences, including reference sequences representing subtypes A-D, F-H, J and K http://hiv-web.lanl.gov, were performed by the CLUSTAL X program [33] followed by manual editing in the BioEdit Sequence Alignment Editor program [34]. Gaps and ambiguous positions were removed from alignment. Positions of crossover sites were defined based on the distribution of informative sites supporting the two incongruent topologies that maximize the χ^2 ^value [35], a method implemented in Simplot.

### Phylogenetic tree analysis

Phylogenetic relationships between the individual sequence types were determined by two methods: the neighbor-joining (NJ) algorithm of MEGA v.4 [36] and the ML of PHYML v.2.4.4 [37]. For NJ, trees were constructed under the maximum composite likelihood substitution model and bootstrap resampling was carried out 1000 times for analysis by the MEGA software. ML phylogenies were constructed using the GTR + I + G substitution model and a BIONJ starting tree. Heuristic tree searches under the ML optimality criterion were performed using the NNI branch-swapping algorithm. The approximate likelihood ratio test (aLRT) based on a Shimodaira-Hasegawa-like procedure was used as a statistical test to calculate branch support. Comparison of tree topologies between subgenomic regions was performed using the algorithm described by Nye et al [38]. Trees were displayed using the program MEGA v.4 package. The nucleotide similarities were estimated using the maximum composite likelihood model implemented by MEGA v.4 software.

### GenBank accession numbers

GenBank accession numbers for the proviral NFLG sequences reported in this study are (06BR_FPS561: HM026455, 07BR_FPS625: HM026456, 07BR_FPS742: HM026457, 07BR_FPS783: HM026458, 07BR_FPS810; HM026459, 07BR_FPS812: HM026460).

## Results

### Recombinant Analysis

**Figure 1:**
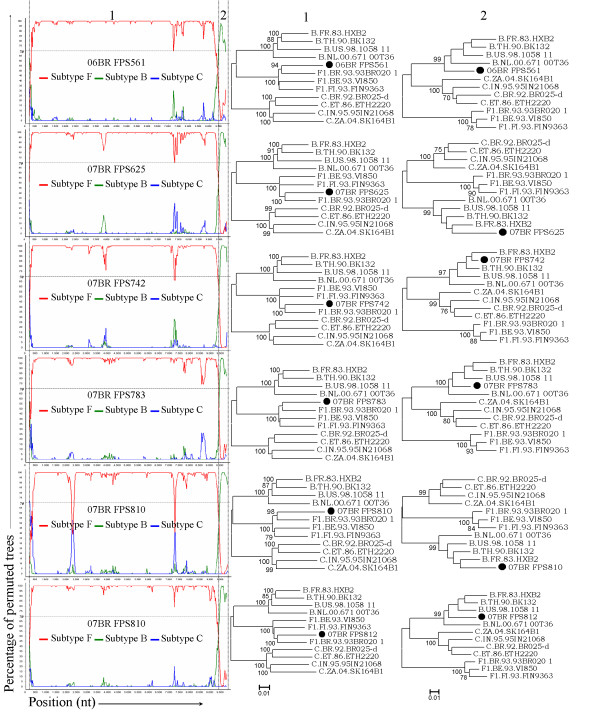
**Phylogenetic relations between the parental regions of our six recombinants**. The left part of the figure shows Bootscan results for the recombinants compared to representatives of HIV-1 subtype B (green line), F1 (red line) C (blue line) reference sequences. The right part refers to the ML phylogenic-based regions between recombination breakpoints as defined by bootscan plot. The recombinants are highlighted with black circles. For clarity purposes, the trees were midpoint rooted. The scale bar represents 0.01 nucleotide substitution per site.

A total of six strains (06BR FPS561, 07BR FPS625, 07BR FPS742, 07BR FPS783, 07BR FPS810, and 07BR FPS812) preliminarily classified as subclade F1 by sequence analysis of a partial *pol *region were corroborated by further phylogenetic analysis of the complete coding sequences and part of the LTR region. Analysis of the proviral NFLGs revealed all isolates retain intact reading frames for a majority of their genes and no gross deletions or rearrangements were observed. The NFLG sequence from each strain was initially investigated using jpHMM which showed them to display identical mosaic structures with a single intersubtype breakpoint identified at the *nef*-U3 overlap (HXB2 position 9347-9365). The recombinant genomes essentially consisted of subclades F1 and B as parental sequences. Fragments identified as subclade F1 were found to cover almost all of the genome coding regions while fragment classified as subtype B consisted of a short sequence comprising the last part of the 3' LTR. Furthermore, the analysis also revealed that all the six isolates had a mosaic sequence pattern nearly identical to the previously published Brazilian BF1 isolates 94BR-RJ-41 (GenBank: AY455781) and 99UFRJ-16 (GenBank: AY455782). Based on these preliminary analyses, we reanalyzed all six sequences using the bootscanning method with three different subtype reference sequences (subtype B, F and C) obtained from the full-length alignment of the HIV sequence database http://hiv-web.lanl.gov. In agreement with the results obtained by jpHMM, bootscanning analysis confirmed similar mosaic structures with almost identical breakpoint positions within these six isolates (Figure 1). The BF1 intersubtype transitions were estimated at nucleotides 9347-9365, based on the HIV HXB2 numbering system, by mapping the informative site and χ^2 ^maximization. To further test for recombination, ML phylogenetic trees were inferred for the regions of nucleotide sequence on either side of the breakpoints detected by bootscan method (Figure 1). This analysis corroborates the results from the bootscan and thus provided unambiguous evidence for a single recombination event supported by high aLRT values among the six isolates.

To rule out the possibility of *Taq*-generated recombinant artifacts, an additional PCR product of 670 bp covering most of the viral LTR was generated in a separate PCR reaction using previously described primers and conditions [29]. The results confirmed the recombination breakpoint obtained using complete viral sequences.

### Phylogenetic analysis of regions bounded by the crossover sites

**Figure 2:**
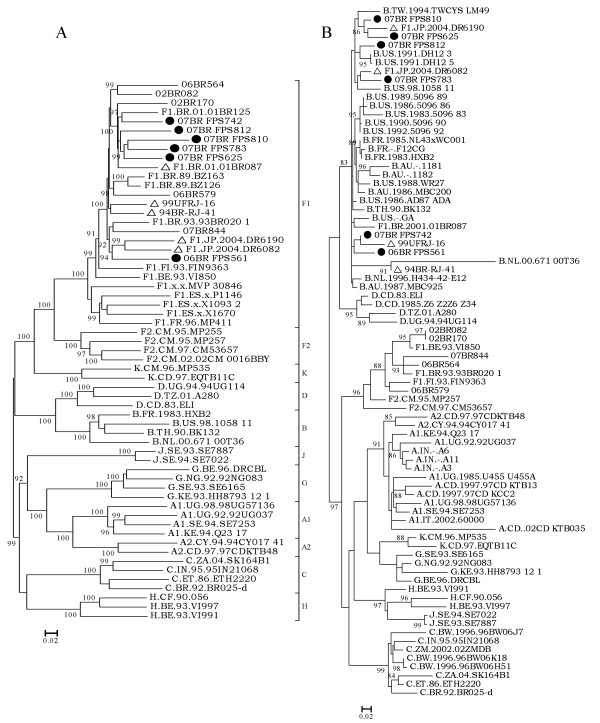
**An exploratory ML tree calculated from fragments between breakpoints of sequences identified in this study (indicated by black circles), published sequences with identical breakpoints (indicated by triangle) and reference sequences of subtype A-D, F-H, J and K http://hiv-web.lanl.gov**. (A) Tree of the viral genomes corresponding to the subclade F1 segments (HXB2 nucleotides 623-9347). (B) Tree of the viral genomes corresponding to the subtype B segments in the LTR region (HXB2 nucleotides 9348-9719). For clarity purposes, the tree was midpoint rooted. The approximate likelihood ratio test (aLRT) values of ≥ 90% are indicated at nodes. The scale bar represents 0.05 nucleotide substitution per site.

**Figure 3:**
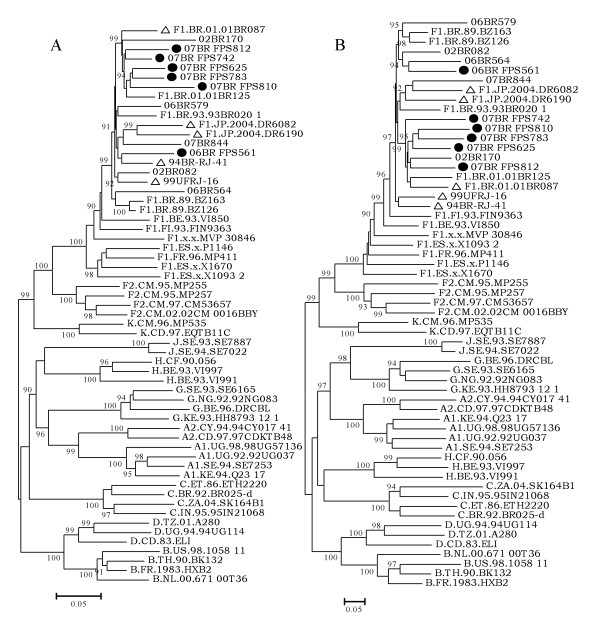
**Maximum likelihood tree of sequences identified in this study (indicated by black circles), published sequences with identical breakpoints (indicated by triangle) and reference strains inferred from full-length *gagpol *(A) and *env *(B) reading frames**. For clarity purposes, the tree was midpoint rooted. The approximate likelihood ratio test (aLRT) values of ≥ 90% are indicated at nodes. The scale bar represents 0.05 nucleotide substitutions per site.

As shown in Figure 2a, phylogenetic reconstructions for F1 specific regions bound by the crossover site, as defined by bootscan analysis, were compared with representatives of all subtype and sub-subtype references available in the HIV database (year 2008) and with other subclade F1 published sequences. The result of the ML tree revealed all our sequences clustered on a branch of subclade F1 and further into one separate sub-branch intrinsic to South America, particularly Brazil (100% aLRT). During analysis of the tree topology of the F1 region depicted in Figure 2a, all new sequences, except isolate 06BR FPS561, formed a single cluster with two previously published Brazilian isolates (F1.BR.01.01BR125 and F1.BR.01.01BR087) supported by 100% aLRT values. Isolate 06BR FPS561 formed a rigid subcluster (94% aLRT) with two strains (F1.JP.2004.DR6190 and F1.JP.2004.DR6082) recently isolated in Japan and believed to be derived from Brazil [Tatsumi et al, unpublished study]. Additionally, the Brazilian isolates 94BR-RJ-41 and 99UFRJ-16 from Rio de Janeiro formed a separate branch (<90% aLRT) distinct from the other branches. To test the stability and branching orders of the F1 fragment in our sequences, ML trees were independently made from *gag-pol *and *env *sequences using the same multiple genome alignment generated for the full length of F1 fragment (Figure 3a &3b). The phylogenetic trees from both regions received an overall topological score of 78.5% according to the algorithm of Nye et al [38]. The computed topological score of the clusters that include all our isolates except 06BR FPS561 in both regions was 100%. Isolate 06BR FPS561 placed the *gag-pol *region within the F1.JP.2004.DR6190, F1.JP.2004.DR6082, 07BR844 and 94BR-RJ-41 cluster with an 84% of aLRT value, while *env *grouped with another subcluster that included 06BR564 and 02BR082 (aLRT 94%). The computed topological score of this cluster in both regions was 30% with a branch length mismatch of 53.5%. Similarly, isolates 94BR-RJ-41 and 99UFRJ-16 changed their topological positions over the *gag-pol and env *regions of their genomes (aLRT <90%). Thus, the shifting of topological positions of isolate 06BR FPS561, 94BR-RJ-41 and 99UFRJ-16 into two different phylogenetic trees is suggestive evidence of intrasubtype recombination event or other factors, such as convergence. Furthermore, the monophyletic cluster of isolates F1.JP.2004.DR6190 and F1.JP.2004.DR6082 depicted in Figure 2a was also supported in trees of both subgenomic regions (Figure 3a &3b).

The phylogenetic tree based on the fragment characterized as subtype B by bootscan from all of the six isolates is shown in Figure 2b. The resulting tree topology agrees with the accepted HIV-1 group M phylogeny and the majority of the internal nodes are supported with high aLRT values. Despite the fact that B fragments in these isolates have shorter sequences and some group M variants cannot resolve some of the internal nodes, all of them can resolve the terminal nodes.

### Molecular rate of CRF46_BF1

Five of the current six BF1 isolates described in this study (designated as CRF46_BF1 in the Los Alamos database) were detected in 36 samples selected from 888 samples infected with HIV-1 F1 based on *pol *subgenomic fragment sequencing [28]. Based on these results, the molecular distribution of the CRF46_BF1 accounts for 0.56% of the HIV-1 circulating strains in São Paulo.

### Identification of Related HIV-1 Strains in the database

A search for similar recombination patterns in a sequence database revealed the occurrence of three isolates from Brazil (GenBank: AY455781; 94BR-RJ-41, AY455782; 99UFRJ-16 and DQ358801; 01BR087) and two isolates from Japan (GenBank: AB480299; F1.JP.2004.DR6082 and AB480301; F1.JP.2004.DR6190). It is to be noted that, as a result of our current analysis, the sequences F1.JP.2004.DR6082, F1.JP.2004.DR6190, and 01BR087, which are characterized as pure subclade F1 [17] [Tatsumi et al, unpublished study], showed strong phylogenetic evidence for recombination among subclade F1 and subtype B, suggesting that a revised classification of these isolates in the GenBank and the HIV databases is appropriate.

**Figure 4:**
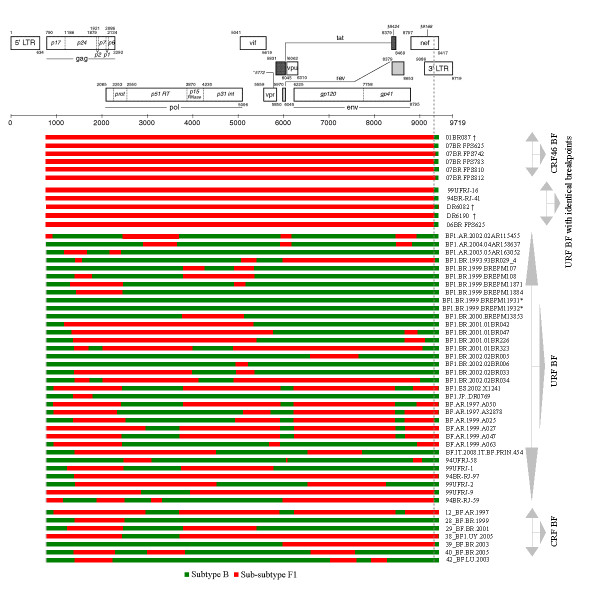
**Schematic representation of the NFLG structure and breakpoint profiles of the sequences identified in this study and other BF1 URF and CRF published sequences**. Sequences marked with the symbol (†) were originally classified as pure F1 subclade. Sequences marked with the symbol (*) were originally classified as pure subtype B. The region of subclade F1 and subtypes B are indicated at the bottom. Positions of breakpoints are marked with grey arrowhead and numbered according to the HXB2 sequence.

Next, we aimed to compare the recombinant profiles of our sequences to other HIV BF1 genomes at the nucleotide level to illustrate the distribution of their breakpoints. This was done by retrieving the full-length genomes from all BF1 and CRF_BF1 isolates available in the Los Alamos database. The automated jpHMM was used for mapping breakpoints with significant recombination signal (Figure 4). Our analysis showed that two variants (GenBank:DQ085869; BREPM11931 and DQ085870; BREPM11931) annotated as BF1 recombinants in the database, appear ancestral to subtype B strains. The recombination mapping of the *nef*-U3 overlap detected in our sequences was also found in CRF39_BF1 and four other URF BF1 recombinants. In addition, most of the sequences have undergone multiple rounds of recombination events. These data suggest that this part of the *nef*-U3 overlap is a possible 'hot spot' for recombination.

Fragment B from all six isolates shared 96% sequence identity with the B stretch in the *nef*-U3 overlap from the Brazilian 93br029 which was isolated in 1993. Thus, we assume that the initial recombination event happened several years before 1993.

### Partial LTR nucleotides alignment features

**Figure 5:**
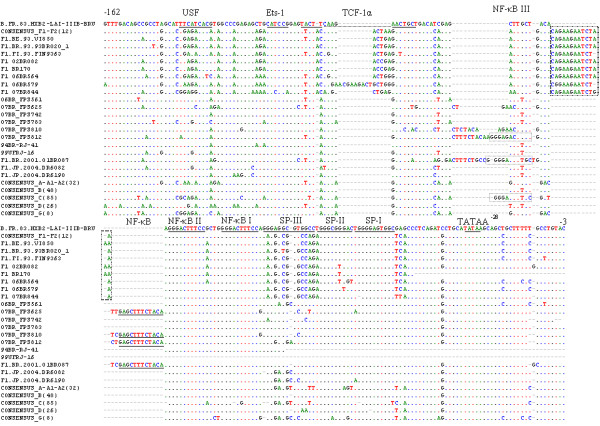
**Alignment of the nucleotide sequences within the LTR region spanning HXB2 positions -162 to +3 (GenBank accession number **K03455**)**. Dots indicate nucleotide identity to the HXB2 sequence and dashes (-) represent gaps introduced to achieve the best alignment. Motifs present in the HXB2 strain are underlined. Boxed sequences in subclade F1 isolates indicate the 13-15 nucleotide insertion.

A detailed scrutinization of the partial nucleotide alignment of the 3' LTR regions relative to HXB2 and consensus sequences of other HIV subtypes (Year 2005) is shown in Figure 5. Conform to the consensus sequence GGGRNNYYCC, additional NF-κB binding sites were found in three strains from the current study. A subclade F1 specific insert of 13-15 [39] nucleotides downstream of the NF-κB^III ^binding site was not observed in our sequences and added further support to our results, indicating that our sequences are not genuine F1 sub-subtypes but BF1 recombinant isolates. Absence of this nucleotide signature was also observed in isolates F1.JP.2004.DR6082, F1.JP.2004.DR6190, and 01BR087, which have previously been classified as pure subclade F1 sequences.

## Discussion

In the present study, we have characterized six NFLG sequences that posses mosaic genomic structure identical to the previously described strains, 94BR_RJ_41 and 99UFRJ_16 with a genome of predominantly subtype F1 and the *nef*-U3 overlap portion of the LTR of subtype B (Figure 1). Moreover, three additional full-length genome sequences, which were initially characterized as pure subclde F1, now clearly appear to harbor a small fragment derived from subtype B in their LTR in a position identical to the breakpoint reported in our sequences. In phylogenetic tree of the full length and subgenomic regions of F1 subclade segment, isolates F1.JP.2004.DR6082 F1.JP.2004.DR6190 (recovered from japanese patients), 94BR-RJ-41 and 99UFRJ-16 (recovered from patients residing in Rio de Janeiro) position outside the single cluster formed by isolates 01BR087 and all BF1 recombinants identified in this study, except 06BR FPS561 (recovered from patients residing in São Paulo) (Figure 2a&3a). The discordant branching between *gag-pol *and *env *sequences of 06BR FPS561, 94BR-RJ-41 and 99UFRJ-16 isolates can be explained by the occurrence of another recombination events after the spread of their common ancestor. Generally, our results suggest that the 11 recombinant sequences were not the result of one, but at least three independent recombination events that produce similar simple recombinant structures. In particular, BF sequences isolated in Japan and Rio de Janeiro may have originated from different BF recombinant ancestors than those sequences isolated in São Paulo. Thus, by excluding all the isolates that branch out of the main cluster, we provide a total of 6 sequences (01BR087 and 5 sequences described in this study) that meet the formal requirement for assigning a new CRF46_BF1. Again, in the phylogenetic tree of the F1 subclade fragment, the two recently isolated Japanese strains (F1.JP.2004.DR6190 and F1.JP.2004.DR6082) formed a rigid subcluster with isolate 06BR FPS561 and branch outside the subcluster formed by the other five viruses described in this study, but still strongly position within the main Brazilian subclade F1 sequences. This result suggests that the viruses found in the Japanese patients share a distinct common ancestry originating in Brazil. It is possible that the heavy traffic of people from both countries across international borders could have facilitated the spread of these viruses in both countries.

Based on the criteria of inclusion of the samples in this study, we were able to show that the CRF46_BF1 accounts for 0.56% of the HIV-1 circulating strains in São Paulo, similar to the frequency of subclade F1 reported from this region [28]. The apparently low prevalence of the CRF46_BF is ecological and may not be due to inherent properties of the virus itself but rather to the chance results of subtype B (a founder virus in Brazil), where it is introduced and consequently established into our HIV infected population before the new CRF and other subtypes are introduced.

Our analysis also showed that the recombination of subclade F1 with subtype B at the *nef*-U3 overlap portion of the LTR appears to be a recurrent finding because it has also been found in CRF39_BF1 and other unique HIV-1 recombinants [17,25,40,41]. In HIV, the existence of recombinational hot spots is common given that they have been described in cell-free systems [42] and exists in the dimer initiation sequence of the HIV-1 5'-untranslated region and some preferential sites across the viral genome [43-46]. Several studies have demonstrated that RNA hairpin structures strongly correlate with recombination hotspots in various regions of the HIV-1 genome[42,43,46,47]. Thus, based on the later mechanisms, it is possible that hairpins promote recombination by hampering the RT during reverse transcription or direct interaction with template [46,48,49].

The HIV-1 LTR region is composed of various cis-acting regulatory components needed for proviral DNA synthesis, integration of the nascent viral cDNA into the host cell genome, transcription and modulation of HIV genes expression [50,51]. Early reports showed that the LTR region is made up of three segments designated as U3, R and U5 [52]. The U3 modulatory region entirely overlaps with *nef *[53] and is essentially required during reverse transcription for first template transfer and integration of the provirus into the host genome. Moreover, this region seems to regulate the transcription pathway of HIV viral promoters by directly or indirectly interacting with a large number of cellular proteins, including NF-AT, Ets-1, USF, AP-1, COUP and Sp1 [54]. Thus, substitution through recombination of the *nef*-U3 overlap portion of the LTR with that of a genetically different subtype, as in our isolates, may affect the binding of both cellular and viral transcription factors. In turn, this may influence viral transcription levels, potentially enhancing the propagation of a recombinant virus leading to the persistance of a circulating form.

Several studies reported successful results in inhibiting HIV-1 replication by using synthetic siRNAs targeting either viral RNA sequences or cellular mRNAs encoding proteins that are critical for HIV-1 replication [55-58]. The study conducted by Yamamoto and his colleagues [59] showed a considerable sustainable suppression of HIV replication and control of CC-chemokine production associated with *nef *expression in HIV-1-infected macrophages following transfection of short hairpin RNA (shRNA) by a lentivirus vector system expressing HIV-specific shRNAs. These results allowed the authors to conclude that lentivirus-vector-based RNA interference of the U3-overlapping region of HIV-1 *nef *may have potential usefulness as a genetic vaccine against HIV-1 infection. Furthermore, Ludwig and collaborator [60] proved that HIV-1 contains an antisense gene in the U3-R regions of the LTR responsible for both an antisense RNA transcript and proteins. This antisense transcript has tremendous potential for intrinsic RNA regulation because of its overlap with the beginning of all HIV-1 sense RNA transcripts by 25 nucleotides. The novel HIV antisense proteins encoded in a region of the LTR that has already been shown to be deleted in some HIV-infected long-term survivors and represent new potential targets for vaccine development [60,61].

Given the biological relevance described to the U3 region, it is probable that the intersubtype recombination in this region could play an important role in HIV evolution with critical consequences for the development of efficient genetic vaccines.

During phylogenetic analysis, the B fragments of our six strains and the other five strains (marked with a triangle symbol in Figure 2b), which showed identical mosaic genomic structures, were clearly distinct from available South American subclade F1 sequences, particularly of Brazilian origin. This result coupled with the absence of the 13-15 nucleotides insertion downstream of the NF-κB^III ^binding site, which is typical for subclade F1, agrees with the interpretation that the segment at the *nef*-U3 overlap portion of the LTR of the eleven isolates originates from subtype B. Unlike the marked clustering of the eleven isolates in the tree generated from the F1 fragment, the tree of fragment B depicted in Figure 2b shows them to fall in different sub-branches within subtype B reference sequences. This result is most likely explained by the short lengths of the fragment B sequences.

## Conclusion

In this study, we describe the NFLG sequence analysis from six HIV-1 isolates sampled from São Paulo and five other published isolates that had an identical breakpoints between subclades F1 and B at the *nef*-U3 overlap portion of LTR. Six of these sequences (five from this study and one from other published sequences) are currently classified as a member of the CRF46_BF1 family. Our data is relevant to guide diagnosis and vaccine development. We conclude that recombination is a potentially important mechanism that significantly contributes to HIV genetic variability with serious implications for diagnosis, drug treatment and optimal vaccine development.

## Competing interests

The authors declare that they have no competing interests.

## Authors' contributions

SS conceived and designed the study, did the data analysis of the sequences, and wrote the manuscript. ÉRP, WKN and VPM conducted the characterization of the full-length genome analysis. ECS designed, wrote the manuscript and directed the study. All authors read and approved the final manuscript.

## References

[B1] Mansky LM The mutation rate of human immunodeficiency virus type 1 is influenced by the vpr gene Virology 1996 222 391 400 10.1006/viro.1996.0436 8806523

[B2] Mansky LM Temin HM Lower in vivo mutation rate of human immunodeficiency virus type 1 than that predicted from the fidelity of purified reverse transcriptase J Virol 1995 69 5087 5094 7541846 10.1128/jvi.69.8.5087-5094.1995 PMC189326

[B3] Perelson AS Neumann AU Markowitz M Leonard JM Ho DD HIV-1 dynamics in vivo: virion clearance rate, infected cell life-span, and viral generation time Science 1996 271 1582 1586 10.1126/science.271.5255.1582 8599114

[B4] Worobey M Holmes EC Evolutionary aspects of recombination in RNA viruses J Gen Virol 1999 80 Pt 10 2535 2543 10573145 10.1099/0022-1317-80-10-2535

[B5] Robertson DL Anderson JP Bradac JA Carr JK Foley B Funkhouser RK Gao F Hahn BH Kalish ML Kuiken C HIV-1 nomenclature proposal Science 2000 288 55 56 10.1126/science.288.5463.55d 10766634

[B6] Gao F Bailes E Robertson DL Chen Y Rodenburg CM Michael SF Cummins LB Arthur LO Peeters M Shaw GM Origin of HIV-1 in the chimpanzee Pan troglodytes troglodytes Nature 1999 397 436 441 10.1038/17130 9989410

[B7] Plantier JC Leoz M Dickerson JE De Oliveira F Cordonnier F Lemee V Damond F Robertson DL Simon F A new human immunodeficiency virus derived from gorillas Nat Med 2009 15 871 872 10.1038/nm.2016 19648927

[B8] Sabino EC Shpaer EG Morgado MG Korber BT Diaz RS Bongertz V Cavalcante S Galvao-Castro B Mullins JI Mayer A Identification of human immunodeficiency virus type 1 envelope genes recombinant between subtypes B and F in two epidemiologically linked individuals from Brazil J Virol 1994 68 6340 6346 8083973 10.1128/jvi.68.10.6340-6346.1994 PMC237055

[B9] Robertson DL Sharp PM McCutchan FE Hahn BH Recombination in HIV-1 Nature 1995 374 124 126 10.1038/374124b0 7877682

[B10] Carr JK Salminen MO Albert J Sanders-Buell E Gotte D Birx DL McCutchan FE Full genome sequences of human immunodeficiency virus type 1 subtypes G and A/G intersubtype recombinants Virology 1998 247 22 31 10.1006/viro.1998.9211 9683568

[B11] Robertson DL Anderson JP Bradac JA Carr JK Foley B Funkhouser RK Gao F BH H Kalish ML Kuiken C Kuiken CL, Foley B, Hahn B, et al HIV-1 nomenclature proposal: a reference guide to HIV-1 classification Human retroviruses and AIDS 1999: a compilation and analysis of nucleic acid and amino acid sequences. Los Alamos, CA 1999 492 505

[B12] Hemelaar J Gouws E Ghys PD Osmanov S Global and regional distribution of HIV-1 genetic subtypes and recombinants in 2004 AIDS 2006 20 W13 23 10.1097/01.aids.0000247564.73009.bc 17053344

[B13] McCutchan FE Global epidemiology of HIV J Med Virol 2006 78 Suppl 1 S7 S12 10.1002/jmv.20599 16622870

[B14] Taylor BS Sobieszczyk ME McCutchan FE Hammer SM The challenge of HIV-1 subtype diversity N Engl J Med 2008 358 1590 1602 10.1056/NEJMra0706737 18403767 PMC2614444

[B15] Barreto CC Nishyia A Araujo LV Ferreira JE Busch MP Sabino EC Trends in antiretroviral drug resistance and clade distributions among HIV-1--infected blood donors in Sao Paulo, Brazil J Acquir Immune Defic Syndr 2006 41 338 341 16540943 10.1097/01.qai.0000199097.88344.50

[B16] Sanabani S Neto WK de Sa Filho DJ Diaz RS Munerato P Janini LM Sabino EC Full-length genome analysis of human immunodeficiency virus type 1 subtype C in Brazil AIDS Res Hum Retroviruses 2006 22 171 176 10.1089/aid.2006.22.171 16478399

[B17] Sanabani S Kleine Neto W Kalmar EM Diaz RS Janini LM Sabino EC Analysis of the near full length genomes of HIV-1 subtypes B, F and BF recombinant from a cohort of 14 patients in Sao Paulo, Brazil Infect Genet Evol 2006 6 368 377 10.1016/j.meegid.2006.01.003 16522378

[B18] Passaes CP Guimaraes ML Bello G Morgado MG Near full-length genome characterization of HIV type 1 unique BC recombinant forms from Southern Brazil AIDS Res Hum Retroviruses 2009 25 1339 1344 10.1089/aid.2009.0167 19954300

[B19] Soares EA Santos RP Pellegrini JA Sprinz E Tanuri A Soares MA Epidemiologic and molecular characterization of human immunodeficiency virus type 1 in southern Brazil J Acquir Immune Defic Syndr 2003 34 520 526 10.1097/00126334-200312150-00012 14657764

[B20] Brindeiro RM Diaz RS Sabino EC Morgado MG Pires IL Brigido L Dantas MC Barreira D Teixeira PR Tanuri A Brazilian Network for HIV Drug Resistance Surveillance (HIV-BResNet): a survey of chronically infected individuals Aids 2003 17 1063 1069 10.1097/00002030-200305020-00016 12700457

[B21] Rodrigues R Scherer LC Oliveira CM Franco HM Sperhacke RD Ferreira JL Castro SM Stella IM Brigido LF Low prevalence of primary antiretroviral resistance mutations and predominance of HIV-1 clade C at polymerase gene in newly diagnosed individuals from south Brazil Virus Res 2006 116 201 207 10.1016/j.virusres.2005.10.004 16332398

[B22] Brennan CA Brites C Bodelle P Golden A Hackett J Jr Holzmayer V Swanson P Vallari A Yamaguchi J Devare S HIV-1 strains identified in Brazilian blood donors: significant prevalence of B/F1 recombinants AIDS Res Hum Retroviruses 2007 23 1434 1441 10.1089/aid.2007.0121 18184087

[B23] Eyer-Silva WA Couto-Fernandez JC Morgado MG Molecular epidemiology of HIV type 1 in inner Rio De Janeiro State, Brazil AIDS Res Hum Retroviruses 2007 23 303 308 10.1089/aid.2006.0199 17331037

[B24] De Sa Filho DJ Sucupira MC Caseiro MM Sabino EC Diaz RS Janini LM Identification of two HIV type 1 circulating recombinant forms in Brazil AIDS Res Hum Retroviruses 2006 22 1 13 10.1089/aid.2006.22.1 16438639

[B25] Guimaraes ML Eyer-Silva WA Couto-Fernandez JC Morgado MG Identification of two new CRF_BF in Rio de Janeiro State, Brazil AIDS 2008 22 433 435 10.1097/QAD.0b013e3282f47ad0 18195572

[B26] Santos AF Sousa TM Soares EA Sanabani S Martinez AM Sprinz E Silveira J Sabino EC Tanuri A Soares MA Characterization of a new circulating recombinant form comprising HIV-1 subtypes C and B in southern Brazil AIDS 2006 20 2011 2019 17053347 10.1097/01.aids.0000247573.95880.db

[B27] Thomson MM Sierra M Tanuri A May S Casado G Manjon N Najera R Analysis of near full-length genome sequences of HIV type 1 BF intersubtype recombinant viruses from Brazil reveals their independent origins and their lack of relationship to CRF12_BF AIDS Res Hum Retroviruses 2004 20 1126 1133 10.1089/aid.2004.20.1126 15585105

[B28] Sanabani SS Pastena ER Kleine Neto W Barreto CC Ferrari KT Kalmar EM Ferreira S Sabino EC Near full-length genome analysis of low prevalent human immunodeficiency virus type 1 subclade F1 in Sao Paulo, Brazil Virol J 2009 6 78 10.1186/1743-422X-6-78 19531216 PMC2704198

[B29] Gao F Robertson DL Morrison SG Hui H Craig S Decker J Fultz PN Girard M Shaw GM Hahn BH Sharp PM The heterosexual human immunodeficiency virus type 1 epidemic in Thailand is caused by an intersubtype (A/E) recombinant of African origin J Virol 1996 70 7013 7029 8794346 10.1128/jvi.70.10.7013-7029.1996 PMC190752

[B30] Schultz AK Zhang M Leitner T Kuiken C Korber B Morgenstern B Stanke M A jumping profile Hidden Markov Model and applications to recombination sites in HIV and HCV genomes BMC Bioinformatics 2006 7 265 10.1186/1471-2105-7-265 16716226 PMC1525204

[B31] Salminen MO Carr JK Burke DS McCutchan FE Identification of breakpoints in intergenotypic recombinants of HIV type 1 by bootscanning AIDS Res Hum Retroviruses 1995 11 1423 1425 10.1089/aid.1995.11.1423 8573403

[B32] Lole KS Bollinger RC Paranjape RS Gadkari D Kulkarni SS Novak NG Ingersoll R Sheppard HW Ray SC Full-length human immunodeficiency virus type 1 genomes from subtype C-infected seroconverters in India, with evidence of intersubtype recombination J Virol 1999 73 152 160 9847317 10.1128/jvi.73.1.152-160.1999 PMC103818

[B33] Thompson JD Gibson TJ Plewniak F Jeanmougin F Higgins DG The CLUSTAL_X windows interface: flexible strategies for multiple sequence alignment aided by quality analysis tools Nucleic Acids Res 1997 25 4876 4882 10.1093/nar/25.24.4876 9396791 PMC147148

[B34] Hall TA BioEdit: a user-friendly biological sequence alignment editor and analysis program for windows 95/98/NT Nucleic Acids SympSer 1999 41 95 98

[B35] Robertson DL Hahn BH Sharp PM Recombination in AIDS viruses J Mol Evol 1995 40 249 259 10.1007/BF00163230 7723052

[B36] Tamura K Dudley J Nei M Kumar S MEGA4: Molecular Evolutionary Genetics Analysis (MEGA) software version 4.0 Mol Biol Evol 2007 24 1596 1599 10.1093/molbev/msm092 17488738

[B37] Anisimova M Gascuel O Approximate likelihood-ratio test for branches: A fast, accurate, and powerful alternative Syst Biol 2006 55 539 552 10.1080/10635150600755453 16785212

[B38] Nye TM Lio P Gilks WR A novel algorithm and web-based tool for comparing two alternative phylogenetic trees Bioinformatics 2006 22 117 119 10.1093/bioinformatics/bti720 16234319

[B39] Jeeninga RE Hoogenkamp M Armand-Ugon M de Baar M Verhoef K Berkhout B Functional differences between the long terminal repeat transcriptional promoters of human immunodeficiency virus type 1 subtypes A through G J Virol 2000 74 3740 3751 10.1128/JVI.74.8.3740-3751.2000 10729149 PMC111883

[B40] Gao F Robertson DL Carruthers CD Morrison SG Jian B Chen Y Barre-Sinoussi F Girard M Srinivasan A Abimiku AG A comprehensive panel of near-full-length clones and reference sequences for non-subtype B isolates of human immunodeficiency virus type 1 J Virol 1998 72 5680 5698 9621027 10.1128/jvi.72.7.5680-5698.1998 PMC110237

[B41] Thomson MM Delgado E Herrero I Villahermosa ML Vazquez-de Parga E Cuevas MT Carmona R Medrano L Perez-Alvarez L Cuevas L Najera R Diversity of mosaic structures and common ancestry of human immunodeficiency virus type 1 BF intersubtype recombinant viruses from Argentina revealed by analysis of near full-length genome sequences J Gen Virol 2002 83 107 119 11752707 10.1099/0022-1317-83-1-107

[B42] Moumen A Polomack L Roques B Buc H Negroni M The HIV-1 repeated sequence R as a robust hot-spot for copy-choice recombination Nucleic Acids Res 2001 29 3814 3821 10.1093/nar/29.18.3814 11557813 PMC55921

[B43] Balakrishnan M Fay PJ Bambara RA The kissing hairpin sequence promotes recombination within the HIV-I 5' leader region J Biol Chem 2001 276 36482 36492 10.1074/jbc.M102860200 11432862

[B44] Balakrishnan M Roques BP Fay PJ Bambara RA Template dimerization promotes an acceptor invasion-induced transfer mechanism during human immunodeficiency virus type 1 minus-strand synthesis J Virol 2003 77 4710 4721 10.1128/JVI.77.8.4710-4721.2003 12663778 PMC152154

[B45] Magiorkinis G Paraskevis D Vandamme AM Magiorkinis E Sypsa V Hatzakis A In vivo characteristics of human immunodeficiency virus type 1 intersubtype recombination: determination of hot spots and correlation with sequence similarity J Gen Virol 2003 84 2715 2722 10.1099/vir.0.19180-0 13679605

[B46] Galetto R Giacomoni V Veron M Negroni M Dissection of a circumscribed recombination hot spot in HIV-1 after a single infectious cycle J Biol Chem 2006 281 2711 2720 10.1074/jbc.M505457200 16291743

[B47] Galli A Lai A Corvasce S Saladini F Riva C Deho L Caramma I Franzetti M Romano L Galli M Recombination analysis and structure prediction show correlation between breakpoint clusters and RNA hairpins in the pol gene of human immunodeficiency virus type 1 unique recombinant forms J Gen Virol 2008 89 3119 3125 10.1099/vir.0.2008/003418-0 19008401

[B48] Moumen A Polomack L Unge T Veron M Buc H Negroni M Evidence for a mechanism of recombination during reverse transcription dependent on the structure of the acceptor RNA J Biol Chem 2003 278 15973 15982 10.1074/jbc.M212306200 12595540

[B49] Roda RH Balakrishnan M Kim JK Roques BP Fay PJ Bambara RA Strand transfer occurs in retroviruses by a pause-initiated two-step mechanism J Biol Chem 2002 277 46900 46911 10.1074/jbc.M208638200 12370183

[B50] Hiebenthal-Millow K Greenough TC Bretttler DB Schindler M Wildum S Sullivan JL Kirchhoff F Alterations in HIV-1 LTR promoter activity during AIDS progression Virology 2003 317 109 118 10.1016/j.virol.2003.08.034 14675629

[B51] Romanchikova N Ivanova V Scheller C Jankevics E Jassoy C Serfling E NFAT transcription factors control HIV-1 expression through a binding site downstream of TAR region Immunobiology 2003 208 361 365 10.1078/0171-2985-00283 14748509

[B52] Varmus H Retroviruses Science 1988 240 1427 1435 10.1126/science.3287617 3287617

[B53] Kirchhoff F Greenough TC Brettler DB Sullivan JL Desrosiers RC Brief report: absence of intact nef sequences in a long-term survivor with nonprogressive HIV-1 infection N Engl J Med 1995 332 228 232 10.1056/NEJM199501263320405 7808489

[B54] Pereira LA Bentley K Peeters A Churchill MJ Deacon NJ A compilation of cellular transcription factor interactions with the HIV-1 LTR promoter Nucleic Acids Res 2000 28 663 668 10.1093/nar/28.3.663 10637316 PMC102541

[B55] Coburn GA Cullen BR Potent and specific inhibition of human immunodeficiency virus type 1 replication by RNA interference J Virol 2002 76 9225 9231 10.1128/JVI.76.18.9225-9231.2002 12186906 PMC136455

[B56] Jacque JM Triques K Stevenson M Modulation of HIV-1 replication by RNA interference Nature 2002 418 435 438 10.1038/nature00896 12087358 PMC9524216

[B57] Novina CD Murray MF Dykxhoorn DM Beresford PJ Riess J Lee SK Collman RG Lieberman J Shankar P Sharp PA siRNA-directed inhibition of HIV-1 infection Nat Med 2002 8 681 686 12042777 10.1038/nm725

[B58] Qin XF An DS Chen IS Baltimore D Inhibiting HIV-1 infection in human T cells by lentiviral-mediated delivery of small interfering RNA against CCR5 Proc Natl Acad Sci USA 2003 100 183 188 10.1073/pnas.232688199 12518064 PMC140921

[B59] Yamamoto T Miyoshi H Yamamoto N Inoue J Tsunetsugu-Yokota Y Lentivirus vectors expressing short hairpin RNAs against the U3-overlapping region of HIV nef inhibit HIV replication and infectivity in primary macrophages Blood 2006 108 3305 3312 10.1182/blood-2006-04-014829 16857988

[B60] Ludwig LB Ambrus JL Jr Krawczyk KA Sharma S Brooks S Hsiao CB Schwartz SA Human Immunodeficiency Virus-Type 1 LTR DNA contains an intrinsic gene producing antisense RNA and protein products Retrovirology 2006 3 80 10.1186/1742-4690-3-80 17090330 PMC1654176

[B61] Deacon NJ Tsykin A Solomon A Smith K Ludford-Menting M Hooker DJ McPhee DA Greenway AL Ellett A Chatfield C Genomic structure of an attenuated quasi species of HIV-1 from a blood transfusion donor and recipients Science 1995 270 988 991 10.1126/science.270.5238.988 7481804

